# Nurses’ perspectives on injection devices for controlled ovarian stimulation in *in-vitro* fertilisation: a cross-sectional survey from the GCC countries

**DOI:** 10.3389/fendo.2025.1664160

**Published:** 2026-01-12

**Authors:** El Samawal Elhakim, Alaa Fouad, Mansour Alorf, Alyah Al Saadoun, Sajida Detho

**Affiliations:** 1 FertiClinic Fertilization Centre, Abu Dhabi, United Arab Emirates; 2 Medical Department, Merck Serono Middle East FZ-LTD, an Affiliate of Merck KGaA, Germany, Dubai, United Arab Emirates; 3 Alorf Hospital, Jahra, Kuwait; 4 Alia International Hospital, Kuwait, Kuwait; 5 Bournhall IVF Centre, Al Ain, United Arab Emirates

**Keywords:** *in vitro* fertilisation, injections, nurses, perspectives, pre-filled pens

## Abstract

**Introduction:**

The design and functionality of injection devices for controlled ovarian stimulation (COS) significantly influence dose accuracy and patient comfort, affecting adherence and treatment success. This study explores the perspectives of couple infertility nurses from the Gulf Council Cooperation (GCC) countries on COS injection devices and their impact on patient experience and adherence.

**Materials and methods:**

A cross-sectional, multicentre, quantitative study was conducted among couple infertility nurses from high- and low-volume assisted reproductive technology (ART) clinics in the United Arab Emirates (UAE) and Kuwait. A structured survey was used to assess nurses’ views on three injection modalities: pre-filled injection pens, syringes/vials, and the COMBO regimen (interchangeable use of pens and vials).

**Results:**

Forty-two nurses were included. The participants perceived the pre-filled pens as easier to use, more convenient, and quicker to teach than syringes. Training time for pre-filled pen use was predominantly less than ten minutes, with 48% requiring under five minutes and 52% requiring under ten minutes. In contrast, training on vial and syringe use was more time-consuming; only 14% reported taking under ten minutes, 55% required 5–10 minutes, and 31% took more than ten minutes. Compared to the COMBO regimen, 96% found the pen easier to teach and faster to train on, and 100% expressed confidence in patients’ ability to self-administer correctly. Satisfaction with the pre-filled pen was high, with 95% and 96% of nurses satisfied or very satisfied when compared to syringes and the COMBO regimen, respectively. A majority (91%) recommended the pen as their first choice or most frequent recommendation. Additionally, nurses perceived lower clinic traffic and fewer errors with the pen, indicating potential economic and safety benefits.

**Conclusion:**

This exploratory, descriptive survey suggests that nurses in couple infertility centres in the GCC region perceive multidose pre-filled pens as easier to use and teach than traditional syringes or COMBO regimens.

## 1 Introduction

Couple infertility represents a significant global public health concern, affecting nearly 17.5% of the adult population worldwide (1). In the Middle East, including the Gulf Cooperation Council (GCC) countries, the burden of couple infertility appears to mirror global trends, with an overall prevalence rate of 17.2% (95% confidence interval [CI] 10.6% to 26.7%) among adults (2). Some studies suggest even a higher prevalence of primary couple infertility among child-seeking women in the Middle East (2.6%) compared to the global estimate (1.9%) (3). In response to the growing burden of couple infertility, there has been a marked rise in the utilisation of assisted reproductive technologies (ART), particularly *in vitro* fertilisation (IVF), in the region (4). Over the past few decades, the demand for IVF has surged across the GCC countries, with a reported market size of ten billion United States Dollars (USD). Additionally, one in six couples was reported to seek access to couple infertility clinics in some GCC countries (5).

Controlled ovarian stimulation (COS) is a fundamental component of IVF protocols aimed at inducing the development of multiple ovarian follicles to maximise the number of retrievable oocytes (6). COS is commonly achieved through the administration of exogenous gonadotropins, primarily recombinant human follicle-stimulating hormone (r-hFSH), delivered via daily subcutaneous injections over a period that may range from 12 to 25 days, depending on the individual’s response and the specific protocol employed (7). However, ovarian response to COS varies considerably among patients and often requires individualisation and dose adjustments to optimise follicular development while minimising the risk of ovarian hyperstimulation syndrome (OHSS) (8). In recent years, there has been a notable shift towards patient self-administration of r-hFSH injections due to convenience and the need for fewer clinic visits (9).

Traditionally, r-FSH has been supplied in vials and administered subcutaneously using single-use syringes (10). While clinically effective, this method presents several practical and psychological challenges for patients undergoing infertility treatment. Concerns about handling needles, accurately measuring doses, and the complexity of preparation steps can all contribute to patient discomfort and anxiety. Many patients express uncertainty over whether the correct dose has been administered or worry about potential dose errors (10–13). These issues—combined with the emotional burden of couple infertility itself— may affect adherence and lead to dropout rates. Consequently, there has been a shift toward user-friendly injection devices, such as pen injectors. Compared to conventional injection techniques, pen injectors offer enhanced accuracy, ease of use, and a more discreet mode of administration, which can contribute to reduced treatment-related stress and improved adherence (14). Additionally, pen injectors simplify the administration process by reducing the required steps and eliminating the need for reconstitution, thereby improving patient confidence and minimising the likelihood of under- or overdosing (7, 15). Furthermore, the design of pen injectors has been associated with lower injection-site adverse events, reduced treatment-related stress, and a lower perceived treatment burden—factors that collectively contribute to improved compliance (14, 16, 17). Recent versions of the pen injectors have been associated with improved dose window visibility, dose confirmation feedback, and small dose increments (as low as 12.5 international units [IU]), allowing for more precise adjustments (11).

couple infertility nurses play a pivotal role in educating patients and their partners on COS protocols, including the correct technique for self-administering injectable r-hFSH. They are often the primary healthcare professionals responsible for training individuals on the use of injection devices. As a result, couple infertility nurses significantly influence patient experience and adherence to injection device use. Their perspectives on the usability of injection devices—particularly the ease of teaching and learning associated with these products—are critical for evaluating patient experience and outcomes. In this cross-section quantitative survey, we evaluated the perspectives of couple infertility nurses from the GCC countries on COS injection devices and their impact on patient experience and adherence.

## 2 Materials and methods

This study was conducted as a market research initiative to explore nurses’ perspectives. In accordance with standard ethical practices for market research, formal ethical committee approval was not required. Participation in the study was entirely voluntary. The first page of the questionnaire clearly outlined the study’s objectives, scope, and the nature of the information being collected. Prior to proceeding with the survey, participants were asked to review this information and provide verbal informed consent to participate. All data were collected anonymously and handled in compliance with applicable data protection regulations. The manuscript was prepared in concordance with the STROBE statement.

### 2.1 Study design and participants

This was a cross-sectional, quantitative, interview-based market research survey conducted between January and March 2025 in the United Arab Emirates (UAE) and Kuwait. Participants were recruited from high (≥500 ART cycles per year) and low (<500 cycles per year) volume ART clinics across the two countries. Eligible participants were couple infertility nurses actively involved in patient education regarding injection devices used in COS. Participants were required to have at least six months of experience in ART services and be willing to provide informed consent. Nurses were recruited using a non-probability convenience sampling across the participating couple infertility centres.

### 2.2 Study questionnaire

Data were collected through face-to-face interviews conducted by trained interviewers using a structured questionnaire to assess nurses’ perspectives on the use of pre-filled, ready-to-use injection pens compared to traditional syringe-based methods for COS. The questionnaire was developed through a multi-phase process, including a comprehensive literature review and clinical expertise. Initially, a focused review of the literature was conducted to identify domains influencing the use of couple infertility injection devices. Data were obtained from peer-reviewed articles, clinical guidelines, and published studies exploring usability, training burden, dosing accuracy, and patient satisfaction with pre-filled pens and traditional syringes. Based on the findings of this review, a preliminary version of the questionnaire was drafted. This draft was subsequently reviewed by a panel of clinical experts, including reproductive endocrinologists, who provided feedback to ensure content validity and practical applicability in ART settings. The revised questionnaire was then pilot-tested with a small group of couple infertility nurses to assess clarity, comprehensiveness, and interpretability. Modifications were made accordingly to develop the final version of the questionnaire.

The final questionnaire consisted of four domains (**Supplementary Table 1**). The first domain captured demographic and professional characteristics, including age, gender, clinical role, years of experience, and monthly patient volume. The second domain compared the attributes of pre-filled, ready-to-use pens to multidose vials via syringes regarding the ease of use and convenience, training requirements and educational burden, dosing confidence and administration accuracy, and the impact of device choice on clinical workflow and efficiency. The third domain examined pre-filled, ready-to-use pens compared to the COMBO regimen (a regimen in which the pens are used for the first half of the IVF cycle and then switched to the multidose syringe in the second half of the cycle) in terms of training demands, dosing accuracy, and patient satisfaction. The second and third domains were evaluated using closed-ended 5-Likert scale (1 = strongly disagree, 5 = strongly agree) questions. The last domain focused on overall satisfaction and clinical preference, including the nurse’s likelihood of recommending a specific injection approach.

### 2.3 Statistical analysis

All statistical analyses were performed using IBM SPSS Statistics for Windows, Version 29.0 (IBM Corp., Armonk, NY, USA). Descriptive statistics were used to summarise participant characteristics and survey responses. Categorical variables were reported as frequencies and percentages, while continuous variables were summarised using means and standard deviations. Responses on Likert scales were analysed both as ordinal variables and, where applicable, dichotomised into “agreement” (e.g., agree/strongly agree) versus “non-agreement” categories for comparative purposes. Because this study was designed as a descriptive survey and was not powered to detect statistical associations or differences between groups, no inferential statistical tests or significance testing were performed.

## 3 Results

A total of 42 nurses participated in the survey. The majority were from the UAE (62%). Regionally, respondents were distributed mainly across Abu Dhabi (31%), Al Jahra (29%), and Dubai (17%). Most participants were between 30 and 39 years of age (64%), followed by those aged 20–29 years (17%) and 40–49 years (17%). Regarding professional experience, the majority had between 1 and 3 years of experience (60%), while 26% had more than 5 years. In terms of clinical exposure, 45% of nurses reported seeing more than 50 patients per month using gonadotropin, while 38% saw between 10 and 50 patients. The nurses reported that, on average, 28% of their patients use self-administered pre-filled pens at home/work, while 20% use them in clinics. On average, 8% of the patients typically use the COMBO regimen. Most nurses (69%) trained more than 10 patients per month on pre-filled pens, while 21% trained 1–3 patients. Training time for pre-filled pen use was predominantly less than ten minutes, with 48% requiring under five minutes and 52% requiring under ten minutes. In contrast, training on vial and syringe use was more time-consuming; only 14% reported taking under ten minutes, 55% required 5–10 minutes, and 31% took more than ten minutes (**Table 1**).

**Table 1:** Characteristics of participating nurses and current practice (N = 42).

Characteristic	n (%)
Country	
United Arab Emirates	26 (62)
Kuwait	16 (38)
Region	
Abu Dhabi	13 (31)
Al Jahra	12 (29)
Dubai	7 (17)
Al Ain	6 (14)
Kuwait City	4 (10)
Age group	
20–29 years	7 (17)
30–39 years	27 (64)
40–49 years	7 (17)
50–60 years	1 (2)
Years of experience	
6–12 months	4 (10)
1–3 years	25 (60)
3–5 years	2 (5)
>5 years	11 (26)
Patients seen per month using gonadotropin	
<10 patients	7 (17)
10–50 patients	16 (38)
>50 patients	19 (45)
Injection scenarios (per month), median % (range)	
Clinic-based syringe administration	13 (0 – 50)
Clinic-based pre-filled pen administration	20 (0 – 60)
Self-administered syringe at home/work	13 (0 – 45)
Self-administered pre-filled pen at home/work	28 (0 – 55)
Combination regimen (pen + syringe)	8 (0 – 50)
Patients trained on pre-filled pens (per month)	
1–3 patients	9 (21)
4–6 patients	1 (2)
7–10 patients	3 (7)
>10 patients	29 (69)
Time to train patient on pre-filled pens	
<5 minutes	20 (48)
5–10 minutes	22 (52)
Time to train patient on vial/syringe use	
<5 minutes	6 (14)
5–10 minutes	23 (55)
>10 minutes	13 (31)

### 3.1 Nurses’ perceptions of multidose pre-filled pens compared to single-use syringes

Nurses reported highly positive perceptions of multidose pre-filled pens compared to single-use syringes across all evaluated domains. In terms of ease of use and convenience, 100% of nurses agreed that the pen is more convenient for patients to carry and use and that it is easier and more user-friendly. A similarly high proportion (100%) reported that patients can inject in a shorter period of time using the pen. Nearly all nurses (97%) felt it was easier for patients to push the injection button, and 98% believed that needle removal and disposal were easy. Under the domain of learning and training, all nurses (100%) found the pen easy to learn and to teach female patients to self-administer ovarian stimulation medications. A total of 91% agreed that it takes less time to teach female patients to use the pen, while 95% believed patients properly understand how to use the pen compared to syringes. The dosing scale on the pen was also considered easier to read by all participants (100%), **Table 2**.

**Table 2:** Nurses’ Perceptions of Attributes of Multidose Pre-filled Pens Compared to Single-Use Syringes (N = 42).

Attribute		Strongly disagree n (%)	Disagree n (%)	Neutral n (%)	Agree n (%)	Strongly agree n (%)
Ease of use and convenience	The patients find the pen more convenient to carry and use at all times.	0 (0)	0 (0)	0 (0)	11 (26)	31 (74)
	The patients find the pen easier to use and more user-friendly.	0 (0)	0 (0)	0 (0)	11 (26)	31 (74)
	Patients can inject in a shorter period of time using the pen.	0 (0)	0 (0)	0 (0)	12 (29)	30 (71)
	It is easier for the patient to push the injection button when injecting.	0 (0)	0 (0)	1 (2)	14 (33)	27 (64)
	It is easy for the patient to remove and discard the needle.	0 (0)	0 (0)	1 (2)	22 (52)	19 (45)
Learning and Training	I found it easy to learn to use the pen.	0 (0)	0 (0)	0 (0)	11 (26)	31 (74)
	I found it easy to teach patients how to use the pen.	0 (0)	0 (0)	0 (0)	13 (32)	29 (68)
	It takes me less time to teach my patients to use the pen.	0 (0)	1 (2)	3 (7)	10 (24)	28 (67)
	Patient properly understands how to use the pen over syringes.	0 (0)	0 (0)	2 (5)	14 (33)	26 (62)
	It is easier to read the dosing scale on the pen.	0 (0)	0 (0)	0 (0)	14 (33)	29 (68)
Confidence and Efficiency	The pen provides me with lower traffic in the clinic and enables me to serve more new patients.	0 (0)	0 (0)	5 (13)	6 (15)	31 (73)
	It is easier to adjust the dose increments with the pen.	0 (0)	0 (0)	2 (5)	12 (29)	28 (67)
	I am confident my patients can regularly administer treatment at the correct dose using this pen.	0 (0)	0 (0)	2 (5)	11 (26)	29 (69)
	I am confident my patients can correctly calculate any top-up dose needed after injection.	0 (0)	0 (0)	1 (2)	14 (34)	26 (63)
	I am confident that my patients can inject the full dose when self-administering at home.	0 (0)	0 (0)	2 (5)	17 (40)	23 (55)
	I find my patients more satisfied with the number of steps involved in preparing/taking the injection.	0 (0)	0 (0)	0 (0)	14 (34)	28 (66)

For confidence and efficiency, 88% of nurses agreed that using the pen reduced clinic traffic and enabled them to see more new patients. The majority found it easier to adjust dose increments (96%) and expressed confidence that patients could regularly administer the correct dose (95%). Confidence in patients’ ability to calculate top-up doses (97%) and inject the full dose at home (95%) was also reported. All nurses (100%) observed greater patient satisfaction with the number of steps involved in preparing and taking the injection (**Table 2**).

### 3.2 Nurses’ perceptions of multidose pre-filled pens compared to the COMBO regimen

Among the 24 nurses who had experience with both multidose pre-filled pens and the COMBO regimen, perceptions strongly favoured the pen. In the learning and training domain, 100% of respondents found the pen easier to learn than the COMBO regimen, while 96% found it easier to teach. A similar majority (96%) agreed it took less time to train patients on the pen. Additionally, 92% believed that patients better understood how to use the pen compared to the COMBO regimen. In terms of confidence and efficiency, 92% of nurses stated that using the pen reduced clinic traffic and enabled them to accommodate more new patients. The same proportion (96%) reported that adjusting dose increments was easier with the pen. Moreover, 100% expressed confidence that patients could regularly administer the correct dose, correctly calculate any top-up doses, and inject the full dose when self-administering at home. Furthermore, 96% of nurses agreed that their patients were more satisfied with the number of steps involved in preparing and taking the injection using the pen compared to the COMBO regimen (**Table 3**).

**Table 3:** Nurses’ Perceptions of Attributes of Multidose Pre-filled Pens Compared to COMBO regimen (N = 24).

Attribute		Strongly disagree n (%)	Disagree n (%)	Neutral n (%)	Agree n (%)	Strongly agree n (%)
Learning and Training	I found it easier to learn to use the pen than the combo regimen.	0 (0)	0 (0)	0 (0)	2 (8)	22 (92)
	I found it easier to teach patients how to use the pen than the combo regimen.	0 (0)	0 (0)	1 (4)	4 (17)	19 (79)
	It takes me less time to teach my patients to use the pen than the combo regimen.	0 (0)	1 (4)	0 (0)	2 (8)	21 (88)
	Patient properly understands how to use the pen over the combo regimen.	0 (0)	0 (0)	2 (8)	5 (21)	17 (71)
Confidence and Efficiency	The pen provides me with lower traffic in the clinic and enables me to serve more new patients.	0 (0)	0 (0)	2 (8)	3 (13)	19 (79)
	It is easier to adjust the dose increments with the pen.	0 (0)	0 (0)	1 (4)	4 (17)	19 (79)
	I am confident my patients can regularly administer treatment at the correct dose using this pen.	0 (0)	0 (0)	0 (0)	6 (25)	18 (75)
	I am confident my patients can correctly calculate any top-up dose needed after injection.	0 (0)	0 (0)	0 (0)	6 (25)	18 (75)
	I am confident that my patients can inject the full dose when self-administering at home.	0 (0)	0 (0)	0 (0)	6 (25)	18 (75)
	I find my patients more satisfied with the number of steps involved in preparing/taking the injection.	0 (0)	0 (0)	1 (4)	4 (17)	19 (79)

### 3.3 Satisfaction with multidose pre-filled pens compared to other injection formats

**Figure 1:**
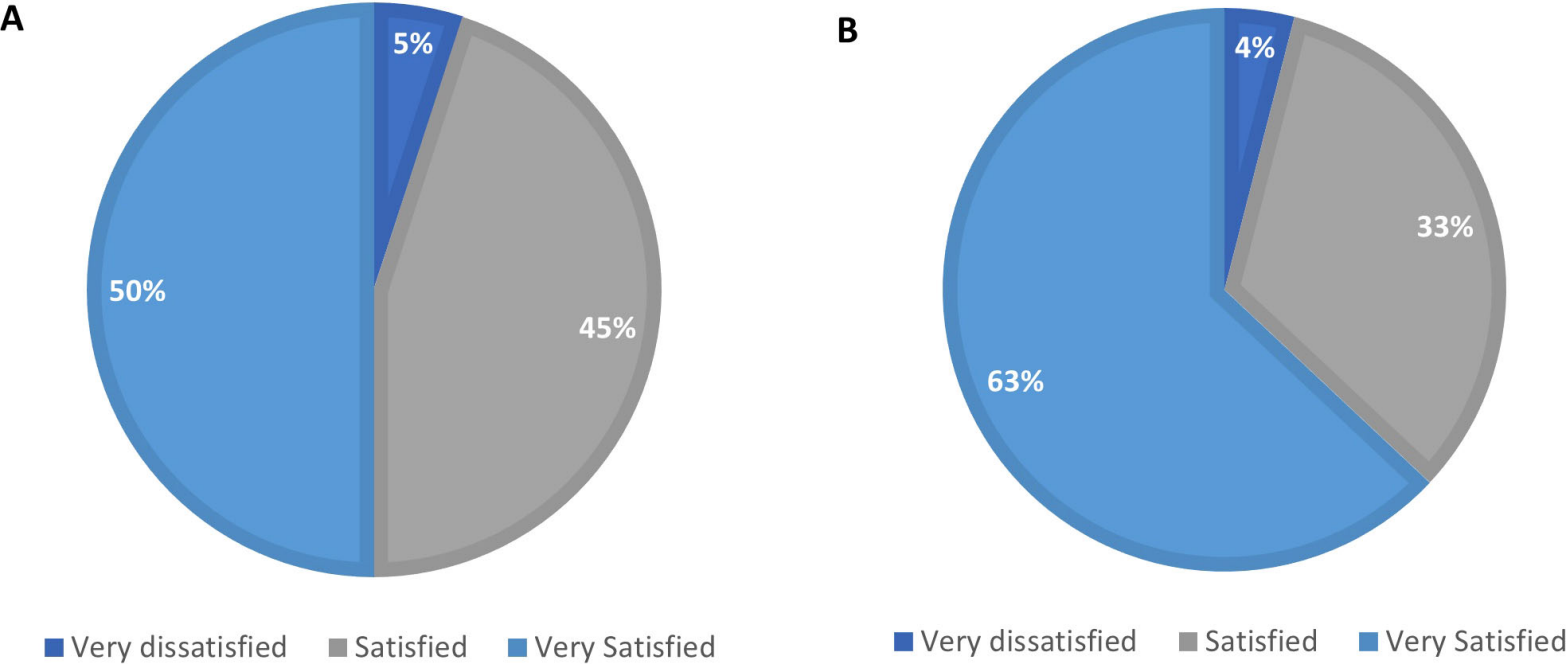
Satisfaction levels with pre-filled, multidose pens compared to single-dose vial **(A)** and COMBO regimen **(B)**.

As shown in **Figure 1**, satisfaction levels with the pre-filled multidose pen were high compared to both the single-dose vial and the COMBO regimen. Compared to the single-dose vial (**Figure 1A**), 95% of nurses reported being either satisfied (50%) or very satisfied (45%) with the pen. Only 5% were very dissatisfied. Satisfaction was even higher when comparing the pen to the COMBO regimen (**Figure 1B**), with 96% of nurses reporting satisfaction—63% were very satisfied, 33% satisfied, and just 4% very dissatisfied.

**Figure 2:**
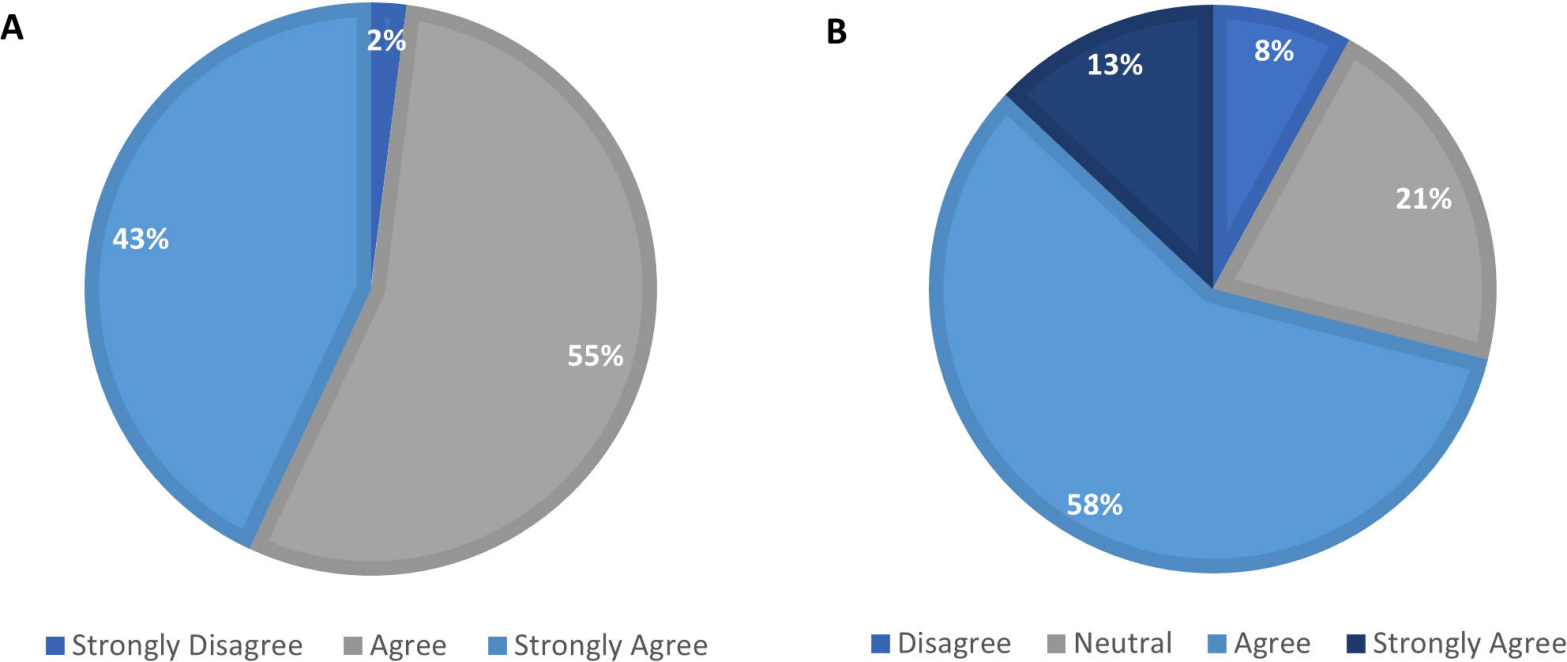
Nurses’ perspectives on the operational efficiency of pre-filled, multidose pens compared to other alternatives **(A)** and higher risk of mistakes associated with the use of COMBO regimen compared to pre-filled multidose pens **(B)**.

## 4 Discussion

The advantages of pre-filled pens over traditional injection formats are well-established across various therapeutic areas (18, 19). However, limited data exist specifically within the context of couple infertility treatment, particularly in the Gulf region. There is a need to bridge this gap by providing real-world perspectives from nurses actively involved in couple infertility care. Thus, the present survey evaluated nurses’ perceptions of the attributes of multidose pre-filled pens for COS compared to single-use syringes and COMBO regimens in couple infertility care across the Gulf region. While previous studies have primarily focused on patient-reported outcomes, this study offers a nursing perspective, given the critical role nurses play in patient education, counselling, and treatment support.

The findings demonstrate a preference for pre-filled pens across multiple domains, including ease of use, training efficiency, patient self-administration, and operational workflow (**Figure 2**). All participating nurses (100%) agreed that the pen was more convenient and easier to use than syringes. Nurses perceived the pre-filled pens as more convenient to carry and use at all times, and patients found it easier to push the injection button and remove and discard the needle. Such findings run in line with previous reports showing that infertility nurses and patients perceived the pre-filled pens as easier to use and more convenient for COS compared to traditional methods (9, 11, 14, 16). Furthermore, studies have shown that even first-time users, specifically women undergoing assisted IVF, were able to learn and use pen injectors effectively, with positive impressions of both the learning experience and the injection process itself (11). This preference can be attributed to ongoing efforts by manufacturers to improve the usability of pen injectors based on real-world user feedback and human factors engineering. Over the years, the design of infertility pen injectors has undergone several enhancements to optimise handling, reliability, and comfort during use (20, 21). For example, the most recent redesigns have focused on improving the clarity of the dose-feedback window, enhancing the grip for easier injection, and increasing the device’s robustness (16). These features help ensure that patients can confidently administer the correct dose and easily identify if any medication remains to be delivered, which is particularly beneficial for self-injecting at home.

Training efficiency emerged as a critical advantage of pre-filled multidose pens in our study. All participating nurses (100%) reported that learning to use the pen was easy and found it easy to teach female patients. In contrast to vial and syringe formats, which required more than ten minutes of training in nearly one-third of cases (31%), training on pen injectors was significantly faster, with 100% of nurses reporting completion in under ten minutes and nearly half (48%) completing it in less than five minutes. This reduction in training time was also well-demonstrated in previous similar studies (9, 11, 14, 16, 22). While our study did not measure workflow or clinic efficiency directly, some nurses felt that devices requiring less instruction may be easier to incorporate into routine practice, particularly in busy settings. Nurses also perceived that the pen format was associated with fewer administration errors and greater patient independence; however, these impressions reflect subjective observations rather than objective error rates or adherence data.

From an economic standpoint, the reduced training burden can translate into meaningful cost savings for clinics and healthcare systems. Fewer training sessions and shorter duration per session can contribute to more efficient resource utilisation (16). Although a formal cost-effectiveness analysis was not performed, the consistent nurse-reported advantages of pre-filled pens highlight their potential to optimise resource use while maintaining or improving patient outcomes. Future economic evaluations should further explore these implications by comparing total costs across different injection modalities, including staff time, error management, and patient adherence.

In addition to improvements in ease of use and training efficiency, nurses perceived that the pre-filled multidose pen is associated with fewer dose administration errors compared to other injection formats. This finding is particularly important in couple infertility treatment, where precise dosing of gonadotropins is essential to optimise outcomes and avoid complications such as under-stimulation or ovarian hyperstimulation (23). One explanation for this perceived safety advantage lies in the iterative design enhancements to pen injectors. Recent updates to the couple infertility pen design have included improvements in the robustness of the device, the ease of handling during use, and the clarity of the dose-feedback window. These upgrades help ensure that users can confidently confirm whether the intended dose has been administered or, if incomplete, identify the remaining amount required (11). Summative usability testing and risk management evaluations have shown that the pre-filled pens can be used reliably to perform all critical tasks associated with dosing (21).

The COMBO regimen, which involves using both vials/syringes and pre-filled pens within a single treatment cycle, remains a common practice in several couple infertility centres across the GCC region. This approach is often driven by formulary preferences, cost considerations, or the need to optimise access to certain gonadotropin brands. While it offers flexibility in product use, the COMBO regimen also introduces complexity for both healthcare providers and patients, particularly when switching between administration techniques within the same cycle. Among the 24 nurses with experience with the COMBO regimen and pre-filled pens in the present study, a strong preference emerged for the pen-only approach. Specifically, 96% of nurses found teaching patients how to use the pen easier than the COMBO regimen, and 96% agreed that training time was shorter with the pen. These findings suggest that the added steps and variability associated with COMBO regimens may complicate patient education and increase the likelihood of misuse or confusion. In addition, 71% of nurses perceived a higher risk of mistakes with the COMBO regimen than with the pre-filled pen. This aligns with existing concerns that switching between injection formats—each with different handling techniques, dosing mechanisms, and storage requirements—can overwhelm patients, particularly those undergoing the emotionally and physically demanding process of assisted reproduction.

To our knowledge, few studies have evaluated nurses’ perspectives on injection devices for COS. However, we acknowledge that the present study has some limitations that should be considered when interpreting the findings. First, the sample size was relatively small (N = 42), with only 24 nurses having experience with both pre-filled pens and the COMBO regimen, which may limit the generalisability of the results. Recruitment was based on convenience sampling, which can additionally limit representativeness and generalisability. Second, the study relied on self-reported data, which may be subject to response bias, particularly social desirability bias, where participants may overstate positive perceptions of pre-filled pens. The survey instrument was developed collaboratively with clinical experts but did not undergo formal psychometric validation, which may limit the reliability and reproducibility of the measures used. Additionally, the study focused on nursing perspectives and did not capture patient-reported outcomes or clinical effectiveness data. Finally, economic implications were inferred rather than directly measured, and future studies should incorporate formal health economic evaluations.

In conclusion, this exploratory, descriptive survey suggests that nurses in couple infertility centres in the GCC region perceive multidose pre-filled pens as easier to use and teach than traditional syringes or COMBO regimens. Nurses perceived the pens as a superior injection method across multiple domains, including ease of use, patient training, operational efficiency, and confidence in patient self-administration. These preferences, while preliminary, highlight the importance of considering nursing experience in decisions related to patient training and device selection. Furthermore, the high levels of patient satisfaction and strong nurse recommendations, particularly as a first-line option, underscore the practical and clinical advantages of adopting multidose pre-filled pens in couple infertility treatment.

## Data Availability

The original contributions presented in the study are included in the article/**Supplementary Material**. Further inquiries can be directed to the corresponding author.
