# Supplementary material for: Nurses’ perspectives on injection devices for controlled ovarian stimulation in in-vitro fertilisation: a cross-sectional survey from the GCC countries
Source: Front Endocrinol (Lausanne). 2026 Jan 12;16:1664160. doi: 10.3389/fendo.2025.1664160 (PMC12832327; doi:10.3389/fendo.2025.1664160)
Supplement: Supplementary file 1 [file Table1.docx]

INTRODUCTION

We are presently carrying out an international market research survey on behalf of a pharmaceutical company on the subject of **Ovarian Stimulation,** and we would like your views on this subject.

In order to not bias the quality of the answers collected during the study, the client's/sponsor's name shall remain confidential.

We would like to reassure you that any information you give will be treated confidentially, without reference to individuals' names. The research conforms to MR Codes of Conduct and any information you give will simply be added to the answers received from others to provide an overall picture of views.

Within the context of this research, we may collect and process your personal data. TRC will comply with all regulations regarding personal data – including European General Data Protection Regulation (GDPR).

We will only collect personal data strictly needed for the research, will implement appropriate technical and organizational measures to ensure the protection of your personal data against unauthorized disclosure or access, will only retain personal data for a limited and fixed period after the end of the research. Your rights towards personal data are:

- the right of access to your personal data;

- the right to rectify your personal data in case of mistakes;

- the right to object processing with your personal data;

- the rights to erasure your personal data;

- the rights to data portability.

For any enquiry regarding the process of your personal data, or to exercise your rights, you can contact the TRC by email [info@researchcorners.com](mailto:info@researchcorners.com) or the Data Protection Officer of TRC by email : [dpo@researchcorners.com](mailto:dpo@researchcorners.com).

This survey is run via a secure server; all information held on this server is protected from external sources.

**By entering the survey link you understand and agree to the following:**

- I understand that this research is sponsored by a pharmaceutical company and is being carried out within the MR Codes of Conduct, i.e. MRS, BHBIA and EphMRA.
- I understand that the aim of this research on the topic of **Ovarian Stimulation** is to gain my views for market research purposes in the development of marketing campaigns, educational materials and the development of new commercial products AND IS NOT INTENDED AS A PROMOTIONAL EXERCISE.
- I understand that the outputs of this research will be used for information only by the sponsoring pharmaceutical company.
- I agree that anything I see or read during this research should be treated as confidential. Any information presented during the course of this research is done so solely to explore reactions to such information and should be assumed to represent hypotheses about what can be said about a product or disease area. It should not be used to influence decisions outside the research setting.
- I understand that the identity of respondents is confidential and none of my details will be passed on to any 3rd party.
- I understand that any information I disclose will be treated in the strictest confidence and the results of the research aggregated to provide an overall picture of attitudes to the areas being covered in this survey. No answers will be attributable to me as an individual.
- I understand that TRC may collect and process my personal data in the conditions stated above.
- I understand that for any enquiry regarding the process of my personal data, or to exercise my rights, I can contact TRC by email : info@researchcorners.com – or the Data Protection Officer of TRC by email : dpo@researchcorners.com.
- You authorize TRC to collect and process your personal data as explained herein.
- You confirm that the transfer of data to TRC, as part of the conduct of this study, complies with the data protection rules for your and your patients' personal data.
- You are free to stop this discussion at any time.
- The time required to fill out this questionnaire is estimated at approximately 25 minutes and this participation on your part will be compensated.

**S1. I confirm all details regarding this research have been duly explained to me; I confirm my agreement with the above terms and conditions including processing with my personal data, and I confirm my participation in this research:**

| 1m | Yes | CONTINUE |
| --- | --- | --- |
| 2m | No | THANK AND CLOSE |

**S.2. You just approved your participation to this research; do you confirm this approval?**

| 1m | Yes | CONTINUE |
| --- | --- | --- |
| 2m | No | THANK AND CLOSE |

SCREENING QUESTIONS

Before we start the interview, we need to ask you some preliminary questions, to confirm that you are the most relevant person to include in our survey.

**Q1. How many years of experience do you have working as: Fertility Nurse / Nurse working in fertility clinic?**

|  | (Single answer) |  |
| --- | --- | --- |
| 1 | Less than 6 months | Terminate |
| 2 | 6-12 months | Continue |
| 3 | 1- 3 years | Continue |
| 4 | >3-5 years | Continue |
| 5 | More than 5 years |  |

Demographics

**Q1. What is your age?**

| …………………….. | Years |
| --- | --- |

**Q2. What is your gender?**

|  | (Single answer) |  |
| --- | --- | --- |
| 1 | Male |  |
| 2 | Female |  |

**Q3. What is your speciality?**

|  | (Single answer) |  |
| --- | --- | --- |
| 1 | Fertility Nurse |  |
| 2 | Nurse working in a fertility clinic |  |
| 3 | Patient educator |  |

**Q4. How many patients do you usually see each month who use gonadotrophin injections for fertility treatments?**

|  | (Single answer) |  |
| --- | --- | --- |
| 1 | Less than 10 |  |
| 2 | 10 to 49 |  |
| 3 | More than 50 |  |

**Q4. What is the number of patients/month with the following injection scenarios for fertility treatment in a typical month in your clinic?**

|  | (Crude number for each item) |  |
| --- | --- | --- |
| 1 | Patients attending the clinic for (powder/solvent for reconstitution) multidose **syringe** administration. | …… |
| 2 | Patients attending the clinic for pre-filled **pen** administration. | …… |
| 3 | Patients self-administering (powder/solvent for reconstitution) multidose **syringe** administration at home/work/etc. | …… |
| 4 | Patients self-administering pre-filled pens at home/work/etc. | …… |
| 5 | Patients using pre-filled **pen for the first half of the IVF cycle and then switch to the multidose syringe in the second half (combo regimen)**. | ……  (If 0, do not ask Q 8) |

**Q6. How many patients do you train in a typical month for the use of injection devices (pre-filled pens) for fertility treatments?**

|  | (Single answer) |  |
| --- | --- | --- |
| 1 | 1 to 3 patients |  |
| 2 | 4 to 6 patients |  |
| 3 | 7 to 10 patients |  |
| 4 | More than 10 patients |  |

Attributes

**Q7.**

**The following is a list of attributes relating to the read-to-use Pen** **compared to (powder/solvent for reconstitution) multidose syringes. Please rate their performance on the following scale.**

1. **Strongly disagree**
2. **Disagree**
3. **Neither nor**
4. **Agree**
5. **Strongly agree**

| **Sector** | **SN** | **Attributes** | **Strongly disagree** | **Disagree** | **Neither nor** | **Agree** | **Strongly agree** | **DK** |
| --- | --- | --- | --- | --- | --- | --- | --- | --- |
| **Ease of Use and Convenience** | **1** | The patients find the pen more convenient to carry and use all times. | **1** | **2** | **3** | **4** | **5** |  |
|  | **2** | The patients find the pen easier to use and more user-friendly | **1** | **2** | **3** | **4** | **5** |  |
|  | **3** | Patients can prepare and inject in a shorter period of time using the pen | **1** | **2** | **3** | **4** | **5** |  |
|  | **4** | It is easier for the patient to push the injection button when injecting | **1** | **2** | **3** | **4** | **5** |  |
|  | **5** | It is easier for the patient to remove and discard the needle | **1** | **2** | **3** | **4** | **5** |  |
| **Learning and Training** | **6** | I found it easier to learn to use the pen. | **1** | **2** | **3** | **4** | **5** |  |
|  | **7** | I found it easier to teach patients how to use the pen. | **1** | **2** | **3** | **4** | **5** |  |
|  | **8** | It takes me less time to teach my patients to use the pen. | **1** | **2** | **3** | **4** | **5** |  |
|  | **9** | Patient properly understands how to use the pen over syringes. | **1** | **2** | **3** | **4** | **5** |  |
|  | **10** | It is easier to read the dosing scale on the pen. | **1** | **2** | **3** | **4** | **5** |  |
| **Confidence and Efficiency** | **11** | The pen provides me with lower traffic in the clinic and enables me to serve more new patients. | **1** | **2** | **3** | **4** | **5** |  |
|  | **12** | It is easier to adjust the dose increments with the pen. | **1** | **2** | **3** | **4** | **5** |  |
|  | **13** | I am confident my patients can regularly administer treatment at the correct dose using this pen. | **1** | **2** | **3** | **4** | **5** |  |
|  | **14** | I am confident my patients can correctly calculate any top-up dose needed after injection. | **1** | **2** | **3** | **4** | **5** |  |
|  | **15** | I am confident that my patients can inject the full dose when self-administering at home | **1** | **2** | **3** | **4** | **5** |  |
|  | **16** | I find my patients more satisfied with the number of steps involved in preparing/taking the injection. | **1** | **2** | **3** | **4** | **5** |  |

**Q8.** **Please rate the performance of patients who use the pen for the first half of the IVF cycle and then switch to the multidose syringe in the second half (combo regimen), compared to those who use the pre-filled pen throughout the entire IVF cycle, on the following scale:**

1. **Strongly disagree**
2. **Disagree**
3. **Neither nor**
4. **Agree**
5. **Strongly agree**

| **Sector** | **SN** | **Attributes** | **Strongly disagree** | **Disagree** | **Neither nor** | **Agree** | **Strongly agree** | **DK** |
| --- | --- | --- | --- | --- | --- | --- | --- | --- |
| **Learning and Training** | **1** | I found it easier to learn to use the pen than the combo regimen. | **1** | **2** | **3** | **4** | **5** |  |
|  | **2** | I found it easier to teach patients how to use the pen than the combo regimen. | **1** | **2** | **3** | **4** | **5** |  |
|  | **3** | It takes me less time to teach my patients to use the pen than the combo regimen. | **1** | **2** | **3** | **4** | **5** |  |
|  | **4** | Patient properly understands how to use the pen over the combo regimen. | **1** | **2** | **3** | **4** | **5** |  |
| **Confidence and Efficiency** | **5** | The pen provides me with lower traffic in the clinic and enables me to serve more new patients than the combo regimen. | **1** | **2** | **3** | **4** | **5** |  |
|  | **6** | It is easier to adjust the dose increments with the pen than with the combo regimen. | **1** | **2** | **3** | **4** | **5** |  |
|  | **7** | I am confident my patients can regularly administer treatment at the correct dose using this pen rather than the combo regimen. | **1** | **2** | **3** | **4** | **5** |  |
|  | **8** | I am confident my patients can correctly calculate any top-up dose needed after injection using the pen rather than the combo regimen. | **1** | **2** | **3** | **4** | **5** |  |
|  | **9** | I am confident that my patients can inject the full dose when self-administering at home using the pen rather than the combo regimen. | **1** | **2** | **3** | **4** | **5** |  |
|  | **10** | I find my patients more satisfied with the number of steps involved in preparing/taking the injection using the pen rather than the combo regimen. | **1** | **2** | **3** | **4** | **5** |  |

Overall Satisfaction

**Q9. To which extent are you satisfied with the Pre-filled Pen over multidose Syringe as an injection mode for the fertility patients?**

**On a scale from 1 to 5 where: 1 means "very dissatisfied" and 5 means "very satisfied"**

| 1 | Very dissatisfied |
| --- | --- |
| 2 | Dissatisfied |
| 3 | Neither nor |
| 4 | Satisfied |
| 5 | Very Satisfied |

**Q10. To which extent are you satisfied with the Pre-filled pens only over Interchangeable use of Pens and Syringes as an injection mode for the fertility patients?**

**On a scale from 1 to 5 where: 1 means "very dissatisfied" and 5 means "very satisfied"**

| 1 | Very dissatisfied |
| --- | --- |
| 2 | Dissatisfied |
| 3 | Neither nor |
| 4 | Satisfied |
| 5 | Very Satisfied |

**Q11. How often do you recommend the use of Pre-filled pens over Interchangeable use of Pens and Syringes for fertility patients?**

| **1** | **I never recommend it** |
| --- | --- |
| **2** | **Recommend it in special circumstances** |
| **3** | **Neutral** |
| **4** | **Recommend it most of the time** |
| **5** | **Highly recommend it / my first preference** |

**Q12. What specific educational tools or resources do you think would enhance the training experience for patients using pre-filled pens?**

|  |
| --- |
|  |
|  |
|  |
|  |
|  |
